# Health Tract, No. 293

**Published:** 1867-05

**Authors:** 


HEALTH TRACT, No. 293.

### CHICKEN AND SMALL POX.

As the very name of the small pox being in a house alarms whole neighborhoods and causes great trouble, anxiety, fear and suffering, intelligent persons should impress on their minds the distinctive difference between the two diseases.

Chicken pox is not preceded by fever, or if by any, it is so very mild as to be scarcely perceptible. Small pox is always attended with decided fever for forty-eight hours before the eruption.

The head-ache of chicken pox is so slight and of such short duration as to give no special inconvenience; in small pox it is always distressing, sometimes causing delirium.

In chicken pox the spots appear on the chest first; in small pox the face first breaks out. The pimples of chicken pox have neither a hardened base nor a central depression; small pox has both.

Chicken pox leaves no pit, and small pox does.

In chicken pox the pimples become filled with a clear fluid the first day of their appearance; and if punctured they subside to a level with the skin; in small pox the pimples are several days in forming, and when they break leave a hard scab which disappears very slowly and leaves a depression in the skin.

Eminent medical men say that chicken pox is not contageous; no one disputes that small pox is so.

In chicken pox the appetite and feeling of wellness generally return in two or three days—certainly within a week; while small pox is seldom recovered from in less than two or three weeks.

In chicken pox the pustules break or subside in a few days, and there is an end of it; in small pox there is secondary fever when the pustules have been matured; about the ninth day the symptoms become aggravated, the skin is hot and dry, tongue white, the pustules become hard and scaly, the pulse increases, and the patient is troubled with tormenting and almost insatiable thirst; and with this secondary fever a variety of symptoms are liable to occur; there is at one time redness like erysipelis or scarlet fever; in others abcesses, boils, carbuncles, &c.; the eyes, abdomen, brain and other parts are liable to be affected; but in chicken pox none of these things ever occur. Small pox attacks persons of all ages, and those who have it over forty seldom recover, while chicken pox is confined to children, and the old do not have it. In small pox the pimples or spots are numerous and round and come out at the same time; in chicken pox they are oval, scattered and ununiform, and appear in successive crops. In chicken pox the pimple if picked becomes a hard pointed scab which falls off and leaves the skin level; in small pox the pimple has a depression in the centre and leaves a depression in the skin. Chicken pox needs no attention but abstinence from meats for two or three days, and to keep the bowels active once every twenty-four hours; small pox requires careful attention and nursing for several weeks. The pimples of chicken pox become filled with a watery fluid in twenty-four hours; those of small pox have a yellow matter after several days.

